# The first three years of screening for medium chain acyl-CoA dehydrogenase deficiency (MCADD) by newborn screening ontario

**DOI:** 10.1186/1471-2431-10-82

**Published:** 2010-11-17

**Authors:** Shelley Kennedy, Beth K Potter, Kumanan Wilson, Lawrence Fisher, Michael Geraghty, Jennifer Milburn, Pranesh Chakraborty

**Affiliations:** 1 Newborn Screening Ontario, Children's Hospital of Eastern Ontario, Ottawa, Ontario, Canada; 2 Department of Epidemiology & Community Medicine, University of Ottawa, Ottawa, Ontario, Canada; 3 Department of Medicine, Ottawa Hospital Research Institute, University of Ottawa, Ottawa, Ontario, Canada; 4 Department of Pediatrics, University of Ottawa, Ottawa, Ontario, Canada; 5 Department of Pathology and Laboratory Medicine, University of Ottawa, Ottawa, Ontario, Canada

## Abstract

**Background:**

Medium chain acyl-CoA dehydrogenase deficiency (MCADD) is a disorder of mitochondrial fatty acid oxidation and is one of the most common inborn errors of metabolism. Identification of MCADD via newborn screening permits the introduction of interventions that can significantly reduce associated morbidity and mortality. This study reports on the first three years of newborn screening for MCADD in Ontario, Canada.

**Methods:**

Newborn Screening Ontario began screening for MCADD in April 2006, by quantification of acylcarnitines (primarily octanoylcarnitine, C8) in dried blood spots using tandem mass spectrometry. Babies with positive screening results were referred to physicians at one of five regional Newborn Screening Treatment Centres, who were responsible for diagnostic evaluation and follow-up care.

**Results:**

From April 2006 through March 2009, approximately 439 000 infants were screened for MCADD in Ontario. Seventy-four infants screened positive, with a median C8 level of 0.68 uM (range 0.33-30.41 uM). Thirty-one of the screen positive infants have been confirmed to have MCADD, while 36 have been confirmed to be unaffected. Screening C8 levels were higher among infants with MCADD (median 8.93 uM) compared to those with false positive results (median 0.47 uM). Molecular testing was available for 29 confirmed cases of MCADD, 15 of whom were homozygous for the common c.985A > G mutation. Infants homozygous for the common mutation tended to have higher C8 levels (median 12.13 uM) relative to compound heterozygotes for c.985A > G and a second detectable mutation (median 2.01 uM). Eight confirmed mutation carriers were identified among infants in the false positive group. The positive predictive value of a screen positive for MCADD was 46%. The estimated birth prevalence of MCADD in Ontario is approximately 1 in 14 000.

**Conclusions:**

The birth prevalence of MCADD and positive predictive value of the screening test were similar to those identified by other newborn screening programs internationally. We observed some evidence of correlation between genotype and biochemical phenotype (C8 levels), and between C8 screening levels and eventual diagnosis. Current research priorities include further examining the relationships among genotype, biochemical phenotype, and clinical phenotype, with the ultimate goal of improving clinical risk prediction in order to provide tailored disease management advice and genetic counselling to families.

## Background

Medium chain acyl-CoA dehydrogenase deficiency (MCADD) is a disorder of mitochondrial fatty acid oxidation and is one of the most common inborn errors of metabolism. The majority of infants with this condition are asymptomatic at birth. However, there is a mortality rate of up to 25% during the first clinical presentation of an undiagnosed infant, which typically occurs between three months and 3 years of age [1,2]. Identification of infants with MCADD via newborn screening, prior to the onset of symptoms, permits the introduction of interventions which can significantly reduce associated morbidity and mortality in affected infants [2].

Infants with MCADD have insufficient activity of the enzyme required to catalyze the first step in the mitochondrial beta-oxidation pathway for medium-chain fatty acids. This process is essential for energy production and also for the formation of ketone bodies in the liver. The latter provides an alternative energy source during periods of increased metabolic demands or reduced dietary intake. When faced with a physiological stress such as an intercurrent illness, infants with MCADD are unable to respond adequately to increased energy demand, which may lead to symptomatic presentation. Symptomatic presentation of MCADD is characterized by an acute metabolic crisis, which typically includes lethargy, vomiting, hypoketotic hypoglycemia, and encephalopathy, and can progress to coma and death. Historically, up to one third of survivors of a metabolic crisis due to MCADD may experience some developmental delay [1].

Infants confirmed to have MCADD are treated with avoidance of fasting and aggressive intervention with intravenous glucose when ill. The prognosis for infants with MCADD is excellent once the diagnosis is established [2,3]. In the published literature, reported deaths after the diagnosis of MCADD have been rare and have mainly occurred in children diagnosed late or in whom early signs were unrecognized and/or intervention was delayed [1,2,4-6].

Infants with undiagnosed MCADD who died prior to a diagnosis being made have often been mislabelled as having had sudden infant death syndrome, Reye syndrome, hepatitis, poisoning or unknown cause of death [1]. Unless metabolic testing was conducted on an autopsy specimen, these infants were never identified in attempts to establish the incidence of MCADD [1]. Newborn screening for MCADD has yielded an international birth prevalence of 1 in 14 600 based on the screening of nearly 8.2 million newborns of mainly European descent [7]. This estimate is about two to three-fold higher than the clinically estimated birth prevalence of MCADD in the absence of screening [1,7] and likely reflects both missed diagnoses of symptomatic cases, and identification of asymptomatic cases through screening that may have never come to clinical attention.

The MCAD protein is produced by the *ACADM *gene (chromosome 1p31). More than 30 mutations in this gene have been identified; most of these are missense mutations whereby one amino acid is substituted for another [1,8]. The most frequent mutation is an adenine to guanine transition at coding position 985 (c.985A > G); this leads to a substitution of glutamate for lysine at position 329 of the protein. About 80% of individuals of European descent who are clinically diagnosed with MCADD are homozygous for the common mutation [1,8]. Newborn screening has revealed a more varied mutation spectrum, with c.985A > G homozygotes representing from 30% to 80% of MCADD cases [1,7].

Biochemically MCADD is characterized by elevated medium-chain acylcarnitines in blood, particularly octanoylcarnitine (C8). It can be identified by screening of dried blood spots via quantitative detection of acylcarnitines using tandem mass (MS/MS) spectrometry. Over the past decade MCADD has been added to the panel of diseases included in newborn screening programs in many regions. Newborn Screening Ontario (NSO) began screening for MCADD on April 5, 2006. Here we review the birth prevalence of MCADD in the Ontario population along with the biochemical and molecular characteristics of confirmed cases and the positive predictive value of the screening test.

## Methods

### Screening methodology

Dried blood spots were sent to Newborn Screening Ontario from submitting centres (primarily hospitals) across the province. The modal age at sample collection was 25 hours and 95% of samples were collected before 4 1/3 days of age. Less than 1% of samples were collected before 24 hours of age and a repeat sample was requested in such cases. Following sample extraction and butylation of amino acids and acylcarnitines, detection and quantitation of acylcarnitine butyl esters was achieved by MS/MS using Waters Quattro Micro tandem mass spectrometers in combination with Waters 1525 μ Binary HPLC Pump using a combination Multiple Reaction Monitoring (MRM) and Parent ion scans of 85 Da.

Screening for MCAD deficiency was accomplished using C8 acylcarnitine (octanoylcarnitine) as a primary analyte with C6 (hexanoylcarnitine), C10 (decanoylcarnitine) and C10:1 (decenoylcarnitine) acylcarnitines as secondary analytes. We also included two ratios in the disorder logic (C8/C10 and C8/C2). The logic and trigger levels used to flag samples as potentially screen positive was initially based on review of the data available in the Region 4 Genetics Collaborative MS/MS data project. From April 2006 through to September 2007, samples that had an elevation of any of the above analytes or ratios were flagged in the information system as a potential positive result, regardless of the concentration of the primary C8 acylcarnitine analyte. The specific logic to flag potential screen positive children was: [C8 > 0.4 OR C6 > 0.24 OR C10:1 > 0.15 OR C10 > 0.3 OR C8/C2 > 0.01 OR C8/C10 > 2.5]. This resulted in a very high number of results requiring manual review during which only those samples with a C8 acylcarnitine level exceeding its cutoff were designated as screen positive. The logic used to flag potential screen positive results was therefore changed in September 2007 to require an elevation of the C8 acylcarnitine thus reducing the number of results being flagged by the information system for review. Using the new logic, samples with a C8 value in excess of 0.5 were flagged as potential screen positive irrespective of the results of any other analytes or ratios, but samples with a C8 value between 0.4 uM and 0.5 uM were flagged only if one or more of the secondary analytes or ratios were also above predetermined cut-off levels (using the same cut-offs specified above), or if the newborn was older than 7 days of age at the time the screening sample was collected. All flagged samples were repunched and re-analyzed in duplicate. Final results with a C8 acylcarnitine ≥ 0.4 μmol/L with accompanying elevations of secondary analytes (or age at collection greater than 7 days), or a C8 acylcarnitine ≥ 0.5 μmol/L in isolation generated a screen positive result. The final decision regarding whether these infants were designated as screen positive was based on the determination of a biochemical geneticist who reviewed all flagged results.

### Diagnostic testing

Positive results were referred to one of five Newborn Screening Treatment Centres across the province of Ontario. These Centres, located at regional Pediatric Academic Health Sciences Centres, are responsible for coordinating diagnostic testing and follow-up care for infants with positive newborn screening results in Ontario. Diagnostic findings are then communicated back to NSO. Criteria for the clinical diagnosis of MCADD have evolved over time. Traditionally, demonstration of deficient enzymatic activity has been considered the gold standard for diagnosis of this condition, however, it is now recognized that the high specificity of a diagnostic plasma acylcarnitine profile, or the demonstration of homozygosity for the common c.985A > G mutation, reduces the need to utilize an enzyme assay for confirmation in a clinical setting [1]. Biochemical, cellular and genotypic characterization of screen positive cases varied amongst the Treatment Centres across the province. In general, infants were classified as truly affected by the responsible metabolic geneticist at each of the Treatment Centres if they were confirmed to be homozygous for the common MCADD mutation or were a compound heterozygote and/or had abnormal plasma acylcarnitines and/or urine organic acids on diagnostic testing. Plasma acylcarnitine profiles were considered abnormal if there was a persistent elevation of C8 acylcarnitine with abnormal C8/C10 ratio. Diagnostic centres did not consistently require the elevation of C10:1 acylcarnitine. Urine organic acids were considered abnormal if characteristic acylglycines were detected or if there was increased excretion of medium chain diacrboxylic acids and their metabolites. Diagnostic centres did not consistently require the detection of hexanoylglycine in the urine as a necessary diagnostic criterion. To be classified as truly affected, it was required that treatment be initiated, and that babies be clinically followed, by a metabolic physician.

## Results

### Screen positive results

From April 5, 2006 to March 31, 2009, an estimated 439 000 infants were screened for MCADD by NSO. Seventy-four infants screened positive. Forty of the screen positives cases were male, 33 were female, and one infant did not have sex specified on the newborn screening requisition (Table 1). Approximately 80% of screen positive infants were born after 37 weeks gestation. The mean birth weight was 2979 grams. Fifteen percent of infants had a birth weight of less than 2500 grams. The majority of infants were reported to be breast fed (75% exclusively, 10% partially) at the time of the newborn screen (Table 1). The median C8 value for screen positive cases was 0.68 uM (mean 4.29 uM, range 0.33-30.41 uM). C8 screening values tended to be lower for screen positive males (median 0.55 uM) compared with females (median 1.40 uM) and for screen positive preterm (median 0.49 uM) compared with screen positive term (0.75 uM) infants (Table 1). The sex difference was statistically significant (p < 0.05, Wilcoxon Rank Sum test). All 74 screen positive infants were referred to one of the five Newborn Screening Treatment Centres for follow-up diagnostic testing and care.

**Table 1:** Characteristics of infants who screened positive for MCADD

	N (percent)	Median C8 (uM)	Mean C8 (uM)
Sex (n = 73)*			
Male	40 (55)	0.55	3.65
Female	33 (45)	1.40	5.18

**Gestational age (n = 61)**			
< = 37 weeks	12 (20)	0.49	3.93
> 37 weeks	49 (80)	0.75	4.09

**Birthweight (n = 65)**			
< 2500 grams	10 (15)	0.49	4.70
> = 2500 grams	55 (85)	0.62	3.70

**Reported feeding status at time of screen (n = 63)**			
Breastfeeding	47 (75)	0.62	3.54
Partial breastfeeding	6 (10)	0.71	3.87
Formula or other (e.g., total parenteral nutrition)	10 (16)	0.94	5.98

* Note: sample sizes differ for sub-sample analyses because of missing data on the blood spot requisition forms (n = 74, median C8 0.68 uM, mean C8 4.29 uM, range 0.33-30.41 uM)

### Follow-up results for screen positive cases

Thirty-one infants have been confirmed to have MCADD and thirty-six infants have been found to be unaffected, based on biochemical and/or molecular testing coordinated at the regional Treatment Centres. The median C8 screening value for confirmed cases of MCADD was 8.93 uM (mean 9.27 uM, range 0.84-30.4 uM), compared to a median C8 screening value of 0.47 uM (mean 0.50 uM, range 0.37-0.79) for infants confirmed to be false positives. Males were overrepresented among false positive cases, although the difference was not statistically significant (p > 0.05, continuity-corrected chi-square test): 69% (25/36) of infants with false positive results but only 45% (14/31) of infants confirmed to have MCADD were male. Results are unavailable for 7 infants who screened positive, either due to death prior to initiation of diagnostic testing (mainly due to complications of prematurity) or because a final diagnosis had not yet been established at the time of this publication.

Molecular testing was performed on 29 of the 31 confirmed cases of MCADD. Molecular results were not provided for 2 of the affected infants. Of the 29 infants with MCADD who had molecular testing, 15 (52%) were homozygous for the common c.985A > G mutation, 5 (17%) were compound heterozygotes for c.985A > G and a second mutation, 5 (17%) were presumed compound heterozygotes for c.985A > G, however a second mutation could not be identified despite clearly abnormal biochemical findings, and 2 (7%) were compound heterozygotes or were homozygous for other abnormal alleles. The remaining 2 infants (7%) had negative results for c.985A > G and no other mutations were reported by the respective Treatment Centres. Molecular testing results were also available for 8 of the infants in the false positive group, who were confirmed to be unaffected mutation carriers; among these infants, 5 were heterozygous for c.985A > G and 3 were heterozygous for other abnormal alleles. These findings are summarized in Table 2 along with the median and mean C8 screening value for each group.

**Table 2:** Median and mean C8 screening value for confirmed cases and confirmed heterozygous carriers of MCADD by genotype

	N	Median Screening C8 (uM)
**Homozygotes: c.985A > G**	15	12.13
		(mean 11.72, range 2.64-30.41)

**Compound heterozygotes: c.985A > G, detectable 2^nd ^mutation**	5	2.01
c.250C > T		(mean 2.82, range 0.84-5.94)
c.388-5G > A^a^		
c.1073A > T^a^		
c.503A > C^a^		
IVS8-13A > G^a^		

**Compound heterozygotes: c.985A > G, no detectable 2^nd ^mutation**	5	2.69
		(mean 6.14, range 0.86-13.69

**Compound heterozygotes or homozygotes for other mutations**	2	12.34
c.799G>A/c.85C > A		(mean 12.34, range 0.88-23.80)
Homozygous for 3 bp deletion c.424-426delAAG^a^		

**Heterozygous mutation carriers**	8	0.50
c.985A > G		(mean 0.52, range 0.44-0.70)
c.347G > A		
c.430-432delAAG^a^		
c.583G > A		

^a^mutation is possibly novel

Based on the experience of Newborn Screening Ontario, the positive predictive value of a screen positive for MCADD was 46.3% (31/67) (excluding unresolved cases). The estimated birth prevalence of this condition in Ontario, based upon 3 years of newborn screening data, is 1 in 14 157.

## Discussion and conclusions

Our analysis of 3 years of data from Newborn Screening Ontario, including approximately 439 000 infants screened since the introduction of universal screening for MCADD, has allowed us to establish the first evidence based birth prevalence rate for MCADD in the province of Ontario. The birth prevalence of 1 in 14 157 live births is in the range of that reported by other newborn screening programs: for example. Rhead reported a birth prevalence of 1 in 14 600 births based on the results of an international survey of newborn screening programs mainly in the US, Northern Europe, and Australia [7]. Grosse and colleagues synthesized estimates from the literature and estimated a frequency of from 1 in 10 000 to 1 in 27 000 among newborn screening populations of European descent, with a lower frequency in populations without European origin [1]. Horvath recently reported a prevalence of 1 in 12 100 births based on the newborn screening program in British Columbia, Canada [9]. We observed a positive predictive value of 46.3% for the screening test used in Ontario. Rhead's survey revealed a wide range of positive predictive values for MCADD screening among newborn screening programs internationally, with the majority of programs reporting positive predictive values of 35% to 100% [7]. The variability is not surprising given that screening algorithms and cut-off values differ considerably among programs.

Fifteen out of the 29 Ontario infants who were confirmed to have MCADD and underwent molecular testing were homozygous for the common mutation (c.985A > G). This represents 52% of the MCADD cases in our population and is consistent with recent published literature suggesting that typically about 40-50% of the population of infants identified with MCADD via newborn screening are homozygous for this mutation [6,10-12], with a range of about 30-80% internationally [1,7]. This compares with estimates that approximately 80% of those identified clinically are homozygous for c.985A > G [1,8]. The C8 screening levels for children with the common mutation in our study were elevated compared to compound heterozygotes with one copy of c.985A > G (both with and without a detectable second mutation). This is also consistent with published data from other newborn screening programs documenting a relationship between genotype and biochemical phenotype, whereby homozygosity for c.985A > G has been associated with more severe levels of biochemical markers relative to some other genotypes [6,7,9,11-13]. There is some additional evidence that individuals with MCADD who are homozygous for c.985A > G may be likely to experience more severe clinical symptoms than those with some other genotypes (i.e., a correlation between genotype and clinical phenotype) [6], but other mutations thought to be equally severe have also been identified [10]. There is considerable variability in clinical presentation even among children who are homozygous for the c.985A > G mutation, highlighting the imperfect relationship between genotype and clinical risk.

Eleven of the MCADD cases in whom mutation analysis was performed were identified as compound heterozygotes, of whom 10 were identified with one copy of the c.985A > G mutation. In 5 of those infants, there was no detectable second mutation. A total of 6 previously unreported mutations were identified among screen positive infants (Table 2). Researchers have used a variety of approaches to try to predict the biochemical impact of rare or novel mutations in the *ACADM *gene detected by newborn screening, including the application of indirect criteria and computer-based modeling techniques to assess the likely functional consequences of particular types of mutations [11-13] and molecular studies to evaluate impact on protein structure and enzyme activity [14,15]. As described, there is also some direct evidence of a relationship between genotype and biochemical phenotype [11,13]. For example, Arnold and colleagues identified a significant association between predicted mutation severity (with severe mutations including c.985A > G as well as deletions, nonsense and splice site mutations) and levels of analytes including C8 and the C8/C2 ratio [10] and Smith and colleagues recently characterized a range of variants of unknown clinical significance according to biochemical parameters [13]. However, it is challenging to extrapolate from these results in order to predict clinical symptoms. For example, the c.199T > C mutation has been widely reported in screen-identified cases of MCADD but not in clinically diagnosed cases, and both in vitro and in vivo studies suggest that it is predicted to be a "mild" mutation; yet it cannot be ruled out as potentially conferring risk of metabolic decompensation [11,12,14]. As noted above, testing for the common c.985A > G was not performed on all screen positive children, nor was sequencing or specific testing for the c.199T > C mutation performed consistently in all presumed positive children. Despite this, and given that sequencing was performed in at least 15 infants of the cohort, it is unusual that no instances of the C.199T > C mutation were detected. Environmental triggers (catabolic stress) are clearly important influences on clinical risk, and some have argued that there must be additional factors (e.g., epigenetic influences, interactions with other pathways) that complicate the relationship between genotype, biochemical phenotype, and clinical presentation [10,11].

Ontario infants confirmed to have MCADD had markedly elevated C8 levels compared to those designated as false positives (median C8 levels of 8.93 uM versus 0.47 uM respectively), including those determined to be heterozygous mutation carriers (median C8 level of 0.5 uM). Eight carriers were identified in the false positive group in our study, for a carrier frequency in that group of at least 22% (8/36), much higher than would be expected in the general population. Other authors have also noted that individuals who are non-affected heterozygous MCADD mutation carriers have higher C8 levels than those with no mutations and they are thus overrepresented among infants with false positive newborn screening results [6,16,17]. Setting a cut-off for disease detection that maximizes sensitivity while minimizing false positive results and the detection of carriers is challenging. There is considerable variation across existing newborn screening programs with respect to the analytes, cut-offs, and algorithms used in screening for MCADD [7]. While there is some evidence that neonatal levels of C8 and other analytes may be predictive of clinical severity among infants diagnosed with MCADD [6,10], again the documented variability in clinical presentation does not allow for precise prediction. Thus, all infants with a confirmed diagnosis of MCADD are considered at risk and require close monitoring and preventive measures to avoid catabolic stress [10].

Finally, a further diagnostic challenge relates to those infants identified with only one mutation and with slightly abnormal biochemical results. This contributes to final results being unavailable for some screen positive infants, as noted above. It is unclear if such infants have a mild form of MCADD or are simply carriers. Enzymology studies are typically considered the gold standard to resolve these cases, however, the results of such studies often fail to provide the desired clarification. Collectively, the challenges we have described in defining and diagnosing MCADD in terms of predicting clinical risk (e.g., with respect to uncertainty of genotype-phenotype correlations, variability in presentation even among known "severe" mutations, and the existence of unresolvable or borderline cases based on marginal biochemistry and an inability to detect two mutations) present barriers to the provision of clear medical guidance and genetic counselling to families. A limitation of the data presented in this paper is the lack of consistency in the biochemical definition of MCADD applied at the various diagnostic centres. While all babies classified in the paper as being truly affected were being treated and followed clinically, the potential that some may not truly be at risk for a metabolic decompensation due to MCADD exists. This clinical judgement must be made with consideration to the available measures to completely rule out risk. It has been proposed that elevation of urinary hexanoylglycine is a necessary diagnostic parameter to confirm a diagnosis of MCADD in screen positive infants [11,13,18]. This was not specifically sought in the majority of infants in this cohort. Of note, 10 of the 15 infants found to be homozygous for the common c.985A > G mutation had qualitative urine organic acids examined. Of these 10, 4 were reported as normal and specific mention of elevated hexanoylglycine was made in only 2 patients; this supports the observation previously reported that routine organic acid analysis may not detect more subtle elevations of hexanoylglycine that may be of clinical significance [19,20]. The harmonization of diagnostic evaluation practices amongst the diagnostic treatment centres is a specific programmatic priority in Ontario. Furthermore, the development of improved clinical risk prediction data for infants with MCADD is a clear research priority.

Additional limitations to this analysis relate to the inability to ascertain whether all identified screen positive individuals include all children with MCADD. While a provincial feedback system currently exists for outside centres to notify NSO of any new MCADD diagnoses that were not identified by screening, the effectiveness of this system remains uncertain. In the absence of this information we cannot provide data on the negative predictive value and the specificity and sensitivity of the screening tests; to date no missed case has been reported. An additional limitation resulting in the incomplete characterization of the screened population relates to the regionalized follow-up model in Ontario. NSO refers screen positive infants to one of five regional Treatment Centres across the province. Variation exists both amongst and within Treatment Centres with regard to i) clinical follow-up testing performed and ii) the level of detail in the results reported back to NSO. A related issue is that while the current clinically driven model of follow-up testing ensures appropriate medical supervision of the infant's diagnostic evaluation, it limits the ability to address a number of relevant research questions. A more standardized provincial approach to follow-up is being considered for MCADD, and other diseases targeted by newborn screening, to ensure consistency in the clinical evaluation of screen positive infants.

Evaluating the long-term effectiveness of newborn screening for MCADD is challenging because simple comparisons of outcomes in screened and unscreened populations do not account for the differences in the spectrum of disease severity. The prevalence of MCADD is higher in screened populations and the distribution of genotypes also differs, suggesting that cohorts of infants identified with MCADD based on clinical symptoms (i.e., metabolic decompensation) include more severe cases than cohorts of infants with MCADD identified by newborn screening. The best available evidence for screening effectiveness to date comes from a large Australian study that compared deaths and severe episodes in screened and unscreened cohorts [2]. The researchers assumed that the actual (rather than detected) prevalence of MCADD in the unscreened group was similar to that observed in the screened group (i.e., they accounted for underdiagnosis in the unscreened population), and they demonstrated that under reasonable assumptions about clinical risk among undetected cases in the unscreened group, newborn screening for MCADD led to a significant reduction in deaths and severe episodes of decompensation [2]. While cost-effectiveness was not evaluated in this study most studies have concluded that screening is cost-effective relative to other health care interventions [21-23]. However, based on the results of the aforementioned Australian cohort study [2], which identified a lower than expected risk of long-term neurologic sequelae among surviving children and a smaller reduction in mortality than has often been assumed based on screening, some previous cost-effectiveness analyses may have overestimated the clinical benefit of newborn MCADD screening [24].

We have described the results of the first three years of newborn screening for MCADD by Newborn Screening Ontario. The birth prevalence of the disease and positive predictive value of the screening test were similar to those identified by other programs internationally. We observed some evidence of correlation between genotype and biochemical phenotype but we faced diagnostic challenges with some cases involving new mutations and/or borderline biochemical results. The clinical effectiveness of newborn screening for MCADD is relatively well established [2]. Current research priorities in the field include further examining the relationships among genotype, biochemical phenotype, and clinical phenotype, with the ultimate goal of improving clinical risk prediction so that disease management advice and genetic counselling can be better tailored to meet the needs of individual families.

## Abbreviations

C8: octanoylcarnitine; MCADD: medium chain acyl-CoA dehydrogenase deficiency; MS/MS: tandem mass spectrometry; NSO: Newborn Screening Ontario

## Competing interests

The authors declare that they have no competing interests.

## Authors' contributions

SK conceived the idea, analysed the data, and prepared the first draft of the manuscript. BKP provided analytical guidance, participated in interpreting the results, and critically revised the manuscript. KW conceived the idea, supervised the study, and critically revised the manuscript. LF drafted the Methods and critically revised the manuscript. MG participated in interpreting the results and critically revised the manuscript. PC supervised the study, interpreted biochemical and molecular data, and critically revised the manuscript. All authors approved the final version of the manuscript.

## Pre-publication history

The pre-publication history for this paper can be accessed here:

http://www.biomedcentral.com/1471-2431/10/82/prepub
